# Inequalities in the distribution of the general practice workforce in England: a practice-level longitudinal analysis

**DOI:** 10.3399/BJGPO.2021.0066

**Published:** 2021-08-18

**Authors:** Claire Nussbaum, Efthalia Massou, Rebecca Fisher, Marcello Morciano, Rachel Harmer, John Ford

**Affiliations:** 1 MPhil Candidate, Department of Public Health and Primary Care, University of Cambridge, Cambridge, UK; 2 Research Associate, Primary Care Unit, Department of Public Health and Primary Care, University of Cambridge, Cambridge, UK; 3 Senior Policy Fellow, GP, The Health Foundation, London, UK; 4 Senior Lecturer, Associate Professor in Health Policy and Economics, Health Organisation, Policy and Economics Research Group, University of Manchester, Manchester, UK; 5 Primary Care Network Co-Clinical Director, GP, East Barnwell Health Centre, Cambridge, UK; 6 Clinical Lecturer in Public Health, Department of Public Health and Primary Care, University of Cambridge, Cambridge, UK

**Keywords:** general practice, health personnel, socioeconomic factors, health services accessibility, social determinants of health

## Abstract

**Background:**

In England, demand for primary care services is increasing and GP shortages are widespread. Recently introduced primary care networks (PCNs) aim to expand the use of additional practice-based roles such as physician associates (PAs), pharmacists, paramedics, and others through financial incentives for recruitment of these roles. Inequalities in general practice, including additional roles, have not been examined in recent years, which is a meaningful gap in the literature. Previous research has found that workforce inequalities are associated with health outcome inequalities.

**Aim:**

To examine recent trends in general practice workforce inequalities.

**Design & setting:**

A longitudinal study using quarterly General Practice Workforce datasets from 2015–2020 in England.

**Method:**

The slope indices of inequality (SIIs) for GPs, nurses, total direct patient care (DPC) staff, PAs, pharmacists, and paramedics per 10 000 patients were calculated quarterly, and plotted over time, with and without adjustment for patient need.

**Results:**

Fewer GPs, total DPC staff, and paramedics per 10 000 patients were employed in more deprived areas. Conversely, more PAs and pharmacists per 10 000 patients were employed in more deprived areas. With the exception of total DPC staff, these observed inequalities widened over time. The unadjusted analysis showed more nurses per 10 000 patients employed in more deprived areas. These values were not significant after adjustment but approached a more equal or pro-poor distribution over time.

**Conclusion:**

Significant workforce inequalities exist and are even increasing for several key general practice roles, with workforce shortages disproportionately affecting more deprived areas. Policy solutions are urgently needed to ensure an equitably distributed workforce and reduce health inequities.

## How this fits in

Pre-existing health inequalities, which are widening owing to COVID-19, may be exacerbated by an inequitably distributed health workforce. In addition, the composition of the general practice workforce is changing with the introduction of PCNs. Here, recent longitudinal trends are explored in the distribution of practice-based staff, including GPs, nurses, and allied health professionals. It is found that significant workforce inequalities exist and are even increasing for several key general practice roles, with workforce shortages disproportionately affecting more deprived areas. Policy solutions are urgently needed to expand access and reduce health inequities.

## Introduction

Health workforce shortages, especially in primary care, have long plagued healthcare systems globally, and the gap between the growing need for services and sufficient staff has been widening.^1–7^ The number of consultations in general practice has increased significantly in recent years, but staff numbers have not kept up with this demand.^5,6,8–12^ The percentage of GPs in the NHS workforce has been steadily decreasing, and the GP workforce is ageing; doctors are increasingly working part-time, which foreshadows future worsening shortages.^5,6,8,12,13^ In 2015, then-Secretary of State for Health Jeremy Hunt promised an additional 5000 GPs for the NHS by 2020, but this was not achieved; instead, it is predicted that there will be a shortage of 7000 GPs by 2024.^5,14^ England’s increasingly depleted primary care workforce will likely be a 'make-or-break' issue for the NHS in coming years.^5^ Evidence suggests that these workforce shortages are not equally distributed across England; areas with higher levels of deprivation tend to have fewer GPs relative to the patient population despite a greater burden of chronic disease.^6,7,10,15–19^ Moreover, GP supply has been linked to health-outcome measures, including mortality rates, self-reported health, and life expectancy, even after controlling for sociodemographic measures.^20–27^

*The NHS Long Term Plan*, published in 2019, emphasised the importance of general practice services and established PCNs, or groups of neighbouring practices that enable economies of scale and integrated care.^28^ PCNs were to boost the primary care workforce through the Additional Roles Reimbursement Scheme (ARRS), which guaranteed financial resources to recruit specified additional roles such as pharmacists, PAs, and paramedics into general practices.^29^ The expanded presence of allied health professionals in primary care is intended to achieve a dual aim of shoring up general practice by reducing pressure on existing clinical staff, as well as delivering outcomes specified in the PCN network contract.

While it is still too early to evaluate recruitment under the ARRS, this research aims to quantify recent trends in the distribution of the general practice workforce, including nurses and additional roles, to uncover any existing workforce inequalities since 2015. It will also examine whether and how the skill mix within practices has changed. This analysis fills a gap in the previous literature on workforce inequalities, which is limited by age (the most recent longitudinal study was published in 2016),^30^ and through an exclusive focus on GPs.^31,32^

## Method

### Data

Quarterly data from September 2015–December 2020 (20 quarters) were obtained from the NHS Digital General Practice Workforce collection.^33^ Practices were matched with their respective PCNs using the NHS Digital GP and GP practice-related data page.^34^

Full-time equivalent (FTE) data were collected on the following staff: GPs, nurses, total DPC staff (all patient-facing staff such as therapists, phlebotomists, dispensers, and additional roles, excluding GPs and nurses), pharmacists, physiotherapists, PAs, occupational therapists, podiatrists, paramedics, social prescribing link workers (SPLWs), and pharmacy technicians. In December 2016 and June 2017, only data on GPs were available. Paramedics were introduced into the dataset in September 2016,^17^ and SPLWs and pharmacy technicians were introduced in June 2019. Atypical practices, that is practices with <750 total patients or <500 patients per GP FTE, were excluded, following previous analyses.^35^ The same method as that described in the General Practice Workforce Data Quality Statement was employed to account for missing workforce data: weighted average estimates were substituted for missing values for GPs, nurses, and total DPC staff in each quarter.^36^

Practice-level Index of Multiple Deprivation (IMD) 2015 and 2019 scores were obtained from the Fingertips National General Practice Profiles.^37^ IMD scores for 2016, 2017, 2018, and 2020 were calculated based on a weighted mean of IMD 2015 and IMD 2019 (75:25, 50:50, 25:75, and 0:100, respectively) and divided into deciles on a scale of 0–1. Deprivation increases with IMD decile; 0.1 corresponds to the least deprived decile, and 1 corresponds to the most deprived decile. Missing IMD data were imputed with IMD scores from the adjacent year, where available.

The Carr-Hill formula was used to adjust for patient need. This resource allocation formula considers patient demographics and health status, list turnover, market forces, and rurality to calculate a weighted practice population.^38,39^ While not an exact measure of patient need,^40^ this formula has been used similarly in previous research,^30^ and is one of the best summary measures available to capture patient need in GP practice populations. Practice-level Carr-Hill data were obtained from the NHS Payments to General Practice collection on NHS Digital, 2015–2016 through 2019–2020, five datasets in total.^41^ Higher Carr-Hill values indicate higher patient need. Missing Carr-Hill data were imputed with values from the adjacent year, where available.

### Analysis methods

The practice-level number of FTE staff per 10 000 patients was calculated for GPs, nurses, total DPC staff, pharmacists, physiotherapists, PAs, occupational therapists, podiatrists, paramedics, SPLWs, and pharmacy technicians. Socioeconomic gradient bar charts depicting the mean FTE staff per 10 000 patients by IMD decile for each workforce variable at each quarter were plotted and visually assessed for linearity. Next, a linear regression analysis of FTE staff per 10 000 patients by IMD decile was conducted at each quarter, with and without Carr-Hill adjustment. The coefficient of this regression is the slope index of inequality (SII).^42^ The SII is a measure of the difference in FTE staff per 10 000 patients between the highest and lowest IMD decile.^30^ A negative SII value corresponds to pro-rich inequality, that is, fewer staff in more deprived deciles; a positive value corresponds to pro-poor inequality, that is, more staff in more deprived deciles. Confidence intervals that overlap zero indicate no significant distributional inequality. These unadjusted and adjusted SIIs were plotted for the quarters with available data from September 2015–December 2020. The SII analysis was also done from March 2019–December 2020 with workforce variables and IMD deciles aggregated to the PCN level for GPs and nurses only, weighted by patient population. Additional roles recruited and employed by PCNs may have been recorded in the newly introduced Primary Care Network Workforce datasets^43^ instead of the General Practice Workforce datasets beginning in March 2020; as a result, PCN-level SII analyses of total DPC staff and each additional role from March 2020 onwards may not be entirely accurate or complete, and were, therefore, excluded from this analysis.

A sensitivity analysis using head count (HC) instead of FTE staff was conducted and compared with the practice-level SII graphs. All analyses were done using Stata (version 16.1).

## Results

### Data overview

Practice populations ranged from 750–89 786 patients. The descriptive statistics, including the percentage of missing data for each workforce variable (FTE staff per 10 000 patients) across the quarters with available data, are shown in Supplementary Table S1.

Occupational therapists, podiatrists, and physiotherapists were excluded owing to insufficient data to detect distributional trends. SPLWs and pharmacy technicians also had low overall numbers but were included in the first step of the analysis because their employment sharply increased in the quarters studied.

The socioeconomic gradient graphs of mean FTE staff per 10 000 patients by IMD decile are included in Supplementary Figure S1. SPLWs and pharmacy technicians were excluded from the regression analyses because they did not show any consistent socioeconomic gradient.

### Workforce inequalities

**Figure 1.**
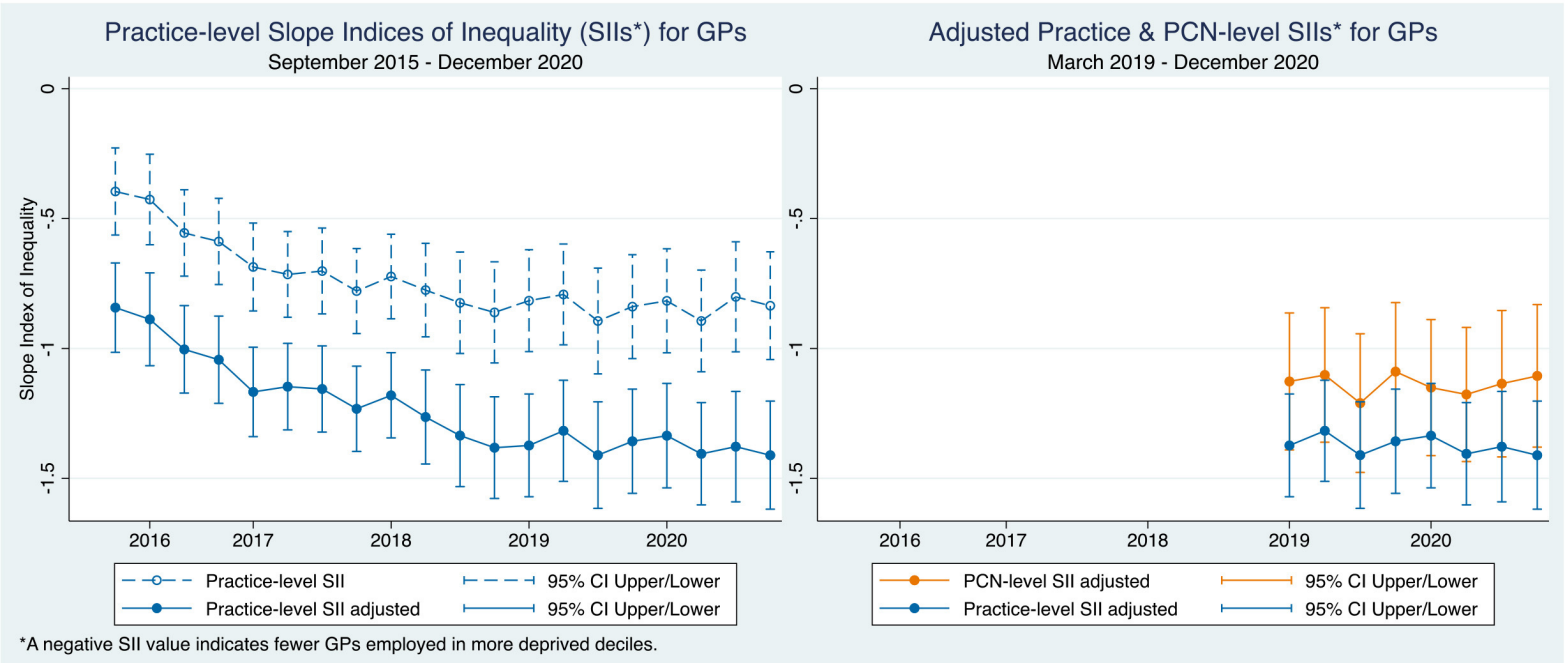
Practice and PCN-level slope indices of inequality (SIIs) over time for GPs. PCN = primary care network

Figure 1 displays the unadjusted and Carr-Hill adjusted SIIs for GPs at each quarter. After Carr-Hill adjustment, the value of the SII tends to decrease (become more negative), indicating higher levels of pro-rich inequality because patient need is often greater in areas with higher deprivation; therefore, to meet demand, more staff are required in more deprived areas.

Across all quarters, there were significantly fewer FTE GPs per 10 000 patients in practices in higher IMD deciles, that is, higher levels of deprivation. This inequality has widened slightly over time, as the SIIs have moved further from the null. While the PCN-level indices for GPs were less negative than the practice-level indices, the SII trends over time appeared similar.

**Figure 2.**
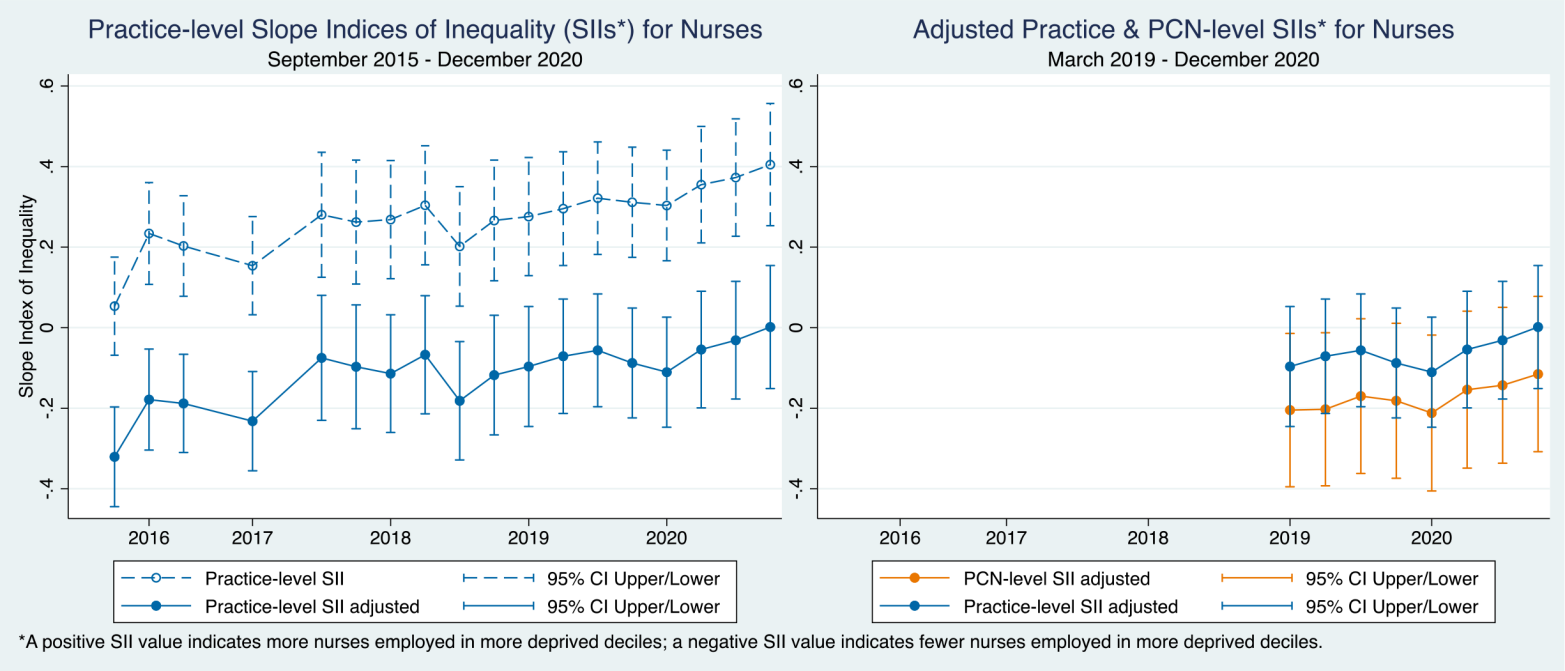
Practice and PCN-level slope indices of inequality (SIIs) over time for nurses. PCN = primary care network

For nurses, the longitudinal trend appears to be moving in the opposite direction, towards a more equal or pro-poor distribution over time. Before adjustment, the positive SII values for nurses suggest that there were more nurses per 10 000 patients employed in more deprived practices (Figure 2); after adjustment, these SIIs became slightly negative, although not statistically significant. The PCN-level SIIs for nurses were more negative than the practice-level SIIs but were only statistically significant (*P*<0.05) in 2019.

**Figure 3.**
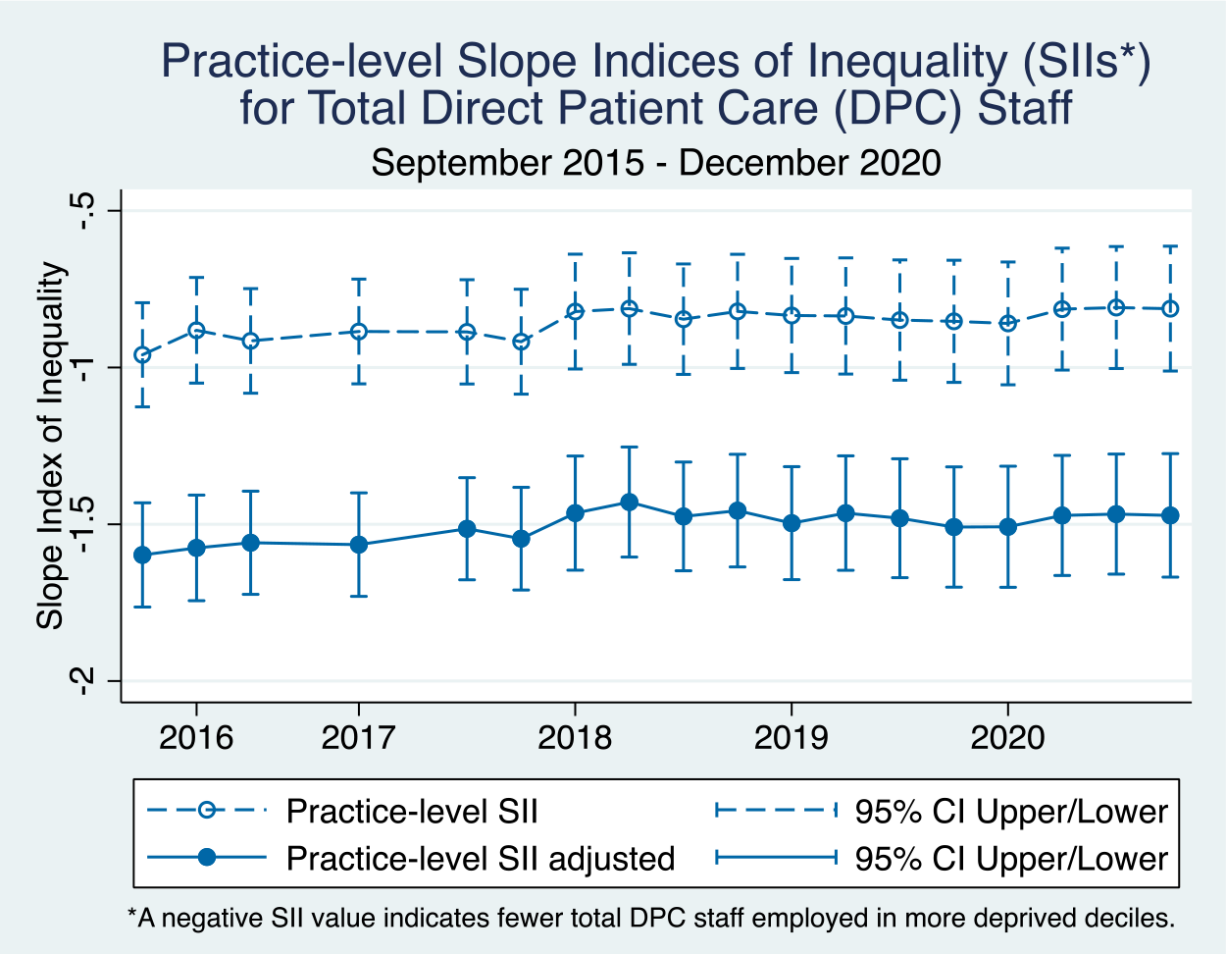
Practice-level slope indices of inequality (SIIs) over time for total direct patient care (DPC) staff

The distribution of total DPC staff mirrors that of GPs, in that there were fewer total DPC staff employed in more deprived areas relative to the patient populations (Figure 3). Similar to Figure 1, the adjusted SII values were significantly further from the null, suggesting higher levels of inequality when accounting for patient need. While the GP SII estimates indicate slightly widening inequality over time, the DPC staff SII estimates show a more stable trend, with persistent but not widening levels of inequality over time.

**Figure 4.**
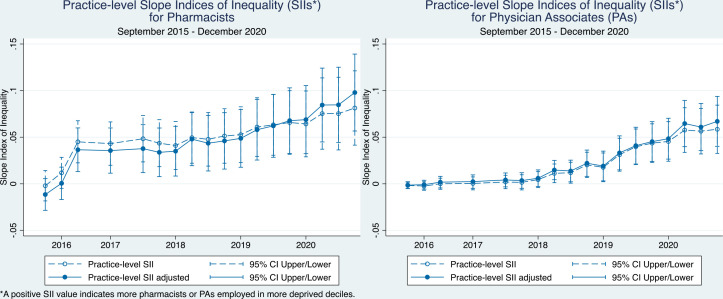
Practice-level slope indices of inequality (SIIs) over time for pharmacists and physician associates (PAs)

For both pharmacists and PAs, a trend of increasing pro-poor inequality was observed at the practice-level, both before and after Carr-Hill adjustment (Figure 4). Starting in September 2016 for pharmacists and June 2018 for PAs, these SII estimates became statistically significant.

**Figure 5.**
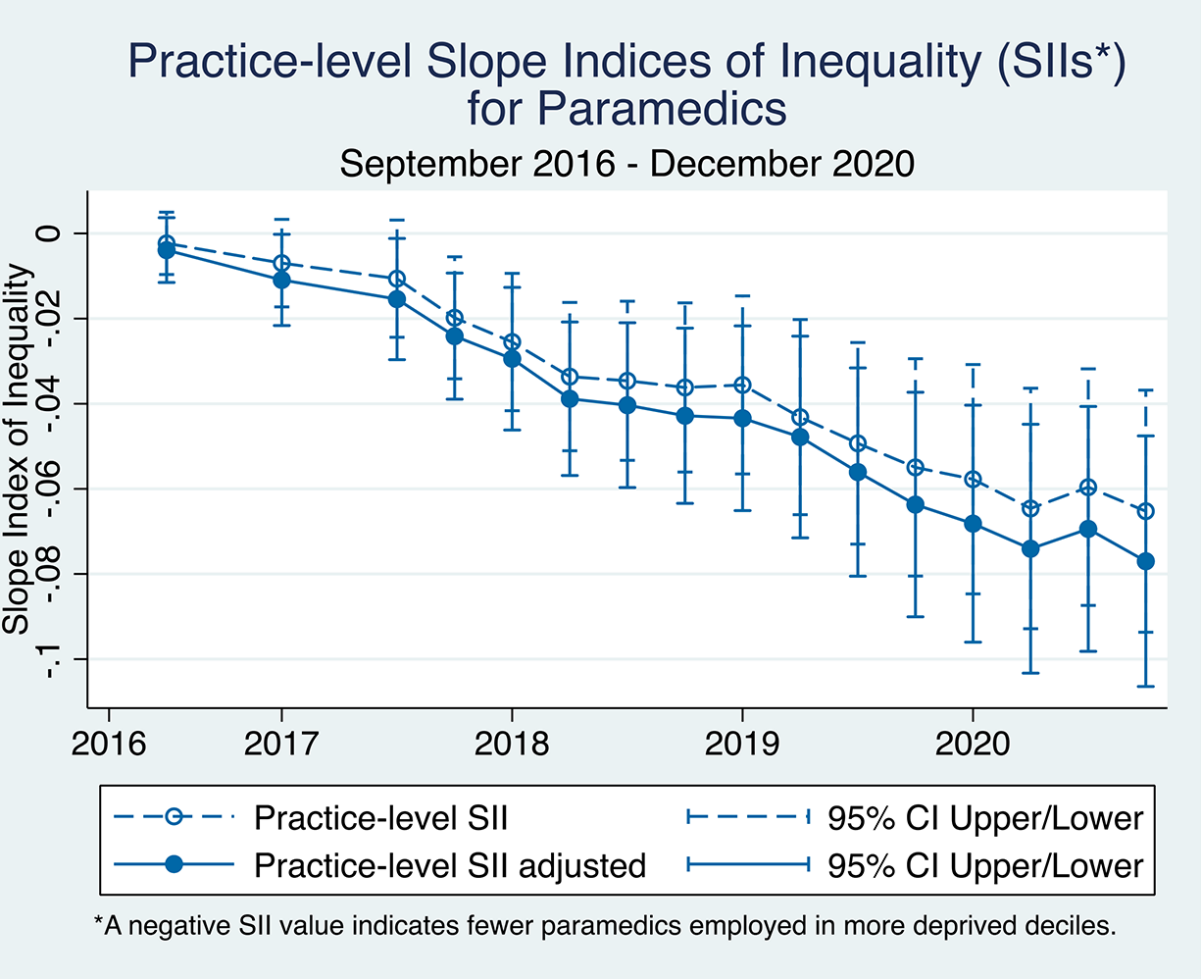
Practice-level slope indices of inequality (SIIs) over time for paramedics

For paramedics, widening pro-rich inequality was observed in both the adjusted and unadjusted practice-level SII estimates (Figure 5). This inequality has been statistically significant since December 2017.

Table 1 summarises the practice-level SIIs for the most recent quarter of workforce data, December 2020. The sensitivity analysis, which is included in Supplementary Figure S2, produced similar results, indicating that the findings are robust to different workforce measures.

**Table 1.** Summary of practice-level results for December 2020

	Adjusted practice-level SII (95% CI)	Difference between the most and least deprived deciles (per 10 000 patients)
FTE	Hours/week
GPs	–1.41^a^ (–1.62 to –1.20)	–1.41	–56.4
Total direct patient care staff	–1.47^a^ (–1.67 to –1.27)	–1.47	–58.8
Nurses	0.0015 (–0.15 to 0.15)	0.0015	0.6
Pharmacists	0.098^a^ (0.057 to 0.14)	0.098	3.9
Physician associates	0.067^a^ (0.040 to 0.094)	0.067	2.7
Paramedics	–0.077^a^ (–0.11 to –0.048)	–0.077	–3.1

^a^ *P*<0.001. FTE = full-time equivalent. SII = slope index of inequality.

## Discussion

### Summary

Inequalities were observed in the distributions of GPs, paramedics, and total DPC staff, with fewer staff employed in more deprived deciles (Table 1). The opposite trend was observed for PAs and pharmacists, with more staff employed in more deprived deciles. For nurses, the unadjusted estimate showed more nurses employed in more deprived deciles; however, the adjusted estimate suggested an approximately equal distribution. These findings suggest that the increasing presence of PAs, pharmacists, and to some extent, nurses, may partially alleviate the undersupply of GPs in more socioeconomically deprived areas. However, the comparison of FTE and hours per week values in Table 1 highlights the limits of this substitution effect. As shown in the hours/week column of Table 1, the relative oversupply of nurses, PAs, and pharmacists in higher IMD deciles only accounted for a total of 7.2 additional hours per week, compared with the observed undersupply of GPs, total DPC staff, and paramedics, which totals to 118.3 fewer hours per week in staffing.

### Strengths and limitations

This analysis extends a previous analysis^30^ on GP inequalities from 2004–2005 to 2013–2014 and updates it to include nurses and additional roles as well as PCN-level indices for GPs and nurses. In addition to its novelty, another strength of this analysis is its exhaustive use of available data. Instead of merging all 20 quarters of data, analyses were run separately at each quarter, minimising the number of excluded practices.

However, this research has several limitations. First, the Carr-Hill formula, which has not been substantively updated since it was first implemented in 2004, has been criticised for not adequately considering the extra pressures that practices in areas of high socioeconomic deprivation face.^2–4,39,40^ As a result, the adjusted SIIs reported may underestimate the true adjusted inequality indices, that is, there may be higher levels of pro-rich inequality than this analysis reports. Furthermore, while SIIs have been used and justified in previous health and workforce inequality analyses,^30,44–46^ the reliance on a crude regression analysis masks variation across and within IMD deciles. The distributions of each workforce variable were assessed visually, and variables that did not show a linear gradient were excluded from further analysis. However, slight non-linear trends are still present in the remaining variables, which can be seen in Supplementary Figure S1. Finally, the publicly available general practice workforce datasets are limited, especially for newly introduced additional roles. Although substitution was employed for missing values where possible, as described in the Method section, the potential for inaccurate or incomplete workforce data as reported by practices remains a limitation of the analysis.

### Comparison with existing literature

The finding that GP workforce shortages disproportionately affect practices in areas of higher deprivation, an inequality that has widened since 2015, is consistent with recent cross-sectional analyses of workforce inequalities.^15–19^ Moreover, the present findings are comparable with a recent longitudinal report by The Health Foundation, which plotted general practice workforce supply over time by IMD quintiles; while this report did not include significance testing, the observed trends align with the results presented in this article.^47^ The most recent longitudinal SII analysis of workforce inequalities reported a slight pro-poor, although not statistically significant, SII estimate in 2013–2014.^30^ However, the SIIs in this analysis had been approaching the null since 2011–2012.^30^ It is, therefore, likely that GP workforce inequality has been increasing since 2011–2012.

This previous longitudinal analysis also found that the primary driver of GP inequality was the opening and closing of practices in more deprived areas, rather than recruitment into existing practices.^30^ Previous reports have shown that practice closures have been increasing since 2013.^48–51^ Likewise, during this study period, far more practices were dropped from the datasets than added, which may have driven the widening inequality observed.

This research demonstrates the existence of an inverse care law (ICL), or a decrease in healthcare delivery with social disadvantage, within general practice in recent years. This is contrasted with previous analyses examining study periods before 2015, which had primarily found what is described by Cookson *et al* ^52^ as a disproportionate care law (DCL), or an increase in healthcare delivery with social disadvantage, but not relative to population need. The finding of an ICL operating for GP supply in England is especially striking given that this form of inequality has primarily been documented in countries with proportionately less or more fragmented public investment in health care; a DCL is more commonly observed in countries with publicly funded health systems that provide universal coverage such as the NHS.^52^

### Implications for research and practice

Reducing health inequalities is a core government commitment and was specified as a primary goal within the *NHS Long Term Plan*.^28,53^ Health needs are often greatest in areas of high socioeconomic deprivation, but we observe a relative undersupply of some key clinical staff in primary care in these areas. One effect of this is the persistence of the ICL in general practice. Addressing this is even more critical in the context of the COVID-19 pandemic, which has disproportionately affected under-resourced and underserved communities, and has widened existing health inequities patterned by class, race, and geography.^54–56^

In England, PCNs are to be tasked with reducing health inequalities. To do this, practices and PCNs in the poorest areas will need relatively more GPs and allied health professionals to respond to the demand for primary care services. The finding — that in general there remains a pro-rich inequality in recruitment of staff in primary care — should be of significant concern to policymakers. They will need to consider why practices and networks in deprived areas are relatively understaffed, and how this can be reversed. Given the significant scale of the proposed expansion of the primary care workforce, policymakers must urgently consider what evidence-based mechanisms can be used to encourage equitable recruitment of additional roles in primary care. This might include but not be limited to incentivisation of DPC posts in understaffed areas, enhanced training offers for these roles, and offering practices and networks in understaffed areas additional recruitment support.^57^ In addition, the impact of substituting nurses and allied health professionals for GPs in under-doctored practices on quality of care and health outcomes is not entirely known. Further research is needed to better understand the effects of skill-mix redesign in the health workforce.

Significant workforce inequalities exist, and are even increasing, for many general practice roles, with some workforce shortages disproportionately affecting more deprived areas. Expanded use of additional roles under the ARRS may partially alleviate GP workload in overstretched practices, but there is a risk that additional workforce will gravitate to more affluent areas, further perpetuating inequity in primary care staffing. These findings underscore the need for more intentional and directed policies addressing workforce inequalities in order to expand access and reduce health inequities.
